# Incidence of myocardial ischemia during treatment with capecitabine: a cohort study with Holter recording and cardiac biomarkers

**DOI:** 10.2340/1651-226X.2025.43089

**Published:** 2025-07-02

**Authors:** Anne Dyhl-Polk, Morten Schou, Kirsten K. Vistisen, Anne-Sophie Sillesen, Stig E. Bojesen, Jens Faber, Merete Vaage-Nilsen, Dorte L. Nielsen

**Affiliations:** a Department of Oncology, Herlev-Gentofte Hospital, University of Copenhagen, Copenhagen, Denmark; b Department of Cardiology, Herlev-Gentofte Hospital, University of Copenhagen, Copenhagen, Denmark; c Faculty of Health and Medical Sciences, University of Copenhagen, Copenhagen, Denmark; d Department of Clinical Biochemistry, Herlev-Gentofte Hospital, University of Copenhagen, Copenhagen, Denmark; e Department of Medicine, Herlev-Gentofte Hospital, University of Copenhagen, Copenhagen, Denmark

**Keywords:** capecitabine, cardiotoxicity, myocardial ischemia, colorectal cancer, breast cancer

## Abstract

**Background and purpose:**

Treatment with fluoropyrimidines can lead to cardiotoxicity. For 5-fluorouracil, silent myocardial ischemia and effort-related myocardial ischemia have been demonstrated. We investigated the incidence of myocardial ischemia and clinical cardiotoxicity during treatment with capecitabine, a pro-drug of 5-fluorouracil.

**Patients and methods:**

We included patients with breast- or colorectal cancer, who received first-time treatment with capecitabine. Holter recording, clinical evaluation, 12-lead electrocardiogram, and measurement of plasma cardiac troponin I and copeptin were performed before and during treatment.

**Results:**

A total of 42 patients with breast cancer and 39 with colorectal cancer were included. Seven patients (9%) experienced clinical cardiotoxicity; five with unstable angina, one with dyspnoea, ST elevations and anterolateral hypokinesia, and one with cardiac arrest. Six patients (8%) had myocardial ischemia on Holter recording during treatment. Among these were two with clinical cardiotoxicity, and four (5.0%) with silent myocardial ischemia. More patients had myocardial ischemia on Holter recording during treatment compared to before, but the difference was not statistically significant (1^st^ cycle: *p* = 0.22, 3^rd^/4^th^ cycle: *p* = 0.50). Plasma copeptin increased during 1^st^ cycle (*p* = 0.004), while cardiac troponin I remained unchanged (*p* = 0.92). More patients had non-sustained ventricular tachycardia during 1^st^ cycle of treatment than before (*p* = 0.020).

**Interpretation:**

Treatment with capecitabine was associated with an incidence of myocardial ischemia of 8%, an incidence of clinical cardiotoxicity of 9%, and an increase in plasma copeptin and the frequency of non-sustained ventricular tachycardia episodes. Increases in cardiac troponin I were rare. The incidence of myocardial ischemia was lower than previously reported for 5-fluorouracil.

## Introduction

Fluoropyridines are cornerstone chemotherapeutics used to treat a wide range of solid tumours. The oral pro-drug capecitabine is used in the treatment of gastrointestinal cancers, cholangiocarcinoma, pancreas-, head and neck- and breast cancer [1–5]. Cardiotoxicity is a well-known side-effect that can be fatal [6–8]. The incidence of capecitabine-induced cardiotoxicity varies from 1.9% to 35.0% in previous studies [6]. The most common manifestation of capecitabine-induced cardiotoxicity is angina with or without myocardial ischemia on electrocardiogram (ECG), followed by arrhythmias, myocardial infarction, cardiac dysfunction, and sudden death [6, 8]. However, two studies examining exercise-induced myocardial ischemia in patients undergoing 5-fluorouracil (5-FU) infusion and capecitabine treatment, respectively, indicated that silent myocardial ischemia occurs with at least the same frequency as angina during a treadmill stress test [9, 10]. Furthermore, we have previously reported that silent myocardial ischemia was present in 15% of patients treated with 5-FU [11].

Cardiac troponins are released into the bloodstream upon myocyte damage and serve as key markers of heart injury [12, 13]. Their diagnostic usefulness is well established in myocardial infarction [14], and cardiac troponins have prognostic value in several cardiac and non-cardiac conditions [15]. Previous small studies conducted on troponin levels during fluoropyrimidine treatment showed no changes in troponin levels during treatment [16–19], but larger sample sizes and advances in troponin assay sensitivity may yield new insights.

Copeptin is the C-terminal part of the vasopressin prohormone and is released from the neurohypophysis upon osmotic, hemodynamic, and stress stimuli [20, 21]. Measurement of copeptin improves the diagnostic accuracy of myocardial infarction compared to troponin alone [22], and elevations in plasma copeptin is a predictor of death in patients with coronary artery disease and patients with heart failure after acute myocardial infarction [23].

We aimed to investigate the incidence of myocardial ischemia during treatment with capecitabine by use of Holter recording, 12-lead ECG, and the cardiac biomarkers, cardiac troponin I (cTnI) and copeptin. Moreover, we evaluated the incidence of clinical events, ventricular tachyarrhythmias and the corrected QT interval (QTc) before and during capecitabine treatment.

## Material and methods

### Patients

Patients with colorectal- or breast cancer treated with capecitabine between February 2013 and June 2017 were consecutively screened for inclusion. Exclusion criteria were age < 18 years, pacemaker or implantable cardioverter defibrillator, prior treatment with fluoropyrimidines, and concurrent treatment with other cardiotoxic agents (ex. lapatinib, trastuzumab or bevacizumab). All participants provided their informed consent.

### Treatment regimens

Patients with colorectal cancer received capecitabine alone or in combination with oxaliplatin or irinotecan. Patients with breast cancer received capecitabine monotherapy. Capecitabine was administered twice daily for 14 days followed by 7 days off. The dosing for each regimen is given in Supplementary Table S1. Dose adjustments were applied according to kidney and liver function, age, haematology, and adverse events.

### Study examinations

Baseline information on cancer disease, antineoplastic treatment, cardiovascular health, and risk factors were obtained from medical records. Further information on cardiovascular health was obtained by use of a questionnaire. The definition of cardiovascular risk factors is provided in Supplementary Table S2.

Prior to and during 1^st^ cycle, as well as before and during 3^rd^/4^th^ cycle, assessment with Holter recording, 12-lead ECG, clinical evaluation, and measurement of cardiac biomarkers were performed. Holter recording was initiated 1–3 days before the commencement of treatment and continued for 4–6 days during treatment (total of 7 days of monitoring). Clinical evaluation, 12-lead ECG, and measurement of cardiac biomarkers were carried out the days the Holter recording was initiated and removed. Patients were instructed to document cardiac symptoms in a diary and to promptly contact the department in case of symptoms. In the event of cardiac symptoms, clinical evaluation, cardiac biomarker measurement, and a 12-lead ECG were performed.

The details about Holter recording and protocol for analyses are described in Dyhl-Polk et al. [11]. In short, we used two-channel Lifecard CF Holter recorders (Delmar Reynolds, Spacelabs Healthcare) to obtain the bipolar leads CM5 and CC5 (Supplementary Figure S1). A trained physician (ADP) conducted semi-manual analyses using Pathfinder SL v1.7 (Spacelabs Healthcare), while an experienced cardiologist (MVN) confirmed episodes of myocardial ischemia or ventricular tachyarrhythmias. Intra-observer variability was good to excellent [11].

12-lead ECGs were obtained with MAC 5500 HD, GE Healthcare, and myocardial ischemia on 12-lead ECG was defined according to guidelines (Supplementary Table S3) [24]. Bazett’s formula was used to estimate the QTc interval. CTnI levels were measured in Li-heparin plasma using the ADVIA Centaur TnI-Ultra assay, Siemens Healthcare Diagnostics Inc (Supplementary Table S3) [12]. Copeptin was measured in ethylenediaminetetraacetic acid (EDTA) plasma utilising the Thermo Scientific^TM^ BRAMS^TM^ Copeptin pro-vasopressin immunofluorescent assay, Thermo Fischer Diagnostics (Supplementary Table S3) [25].

### Endpoints

The primary endpoint was myocardial ischemia on Holter recording or 12-lead ECG. Myocardial ischemia on Holter recording was defined as ST segment (ST) elevations (≥1 mV measured in the J-point) or ST depressions (down-sloped/horizontal, ≥1 mV measured 60 ms after the J-point), lasting at least 1 min. Registration of new episodes was preceded by at least 1-min intervals without ST deviations (Supplementary Table S3). Secondary endpoints were clinical events, elevations and fluctuations in cTnI, ventricular tachyarrhythmias, and QTc interval prolongation (for definitions see Supplementary Table S3). We defined a combined endpoint of myocardial ischemia and clinical events.

The ischemic burden was quantified by multiplying the amplitude and duration of ST-deviations in the channel with most pronounced ischemia. In addition, we recorded the duration of ischemic episodes per day and number of ST-elevation and ST-depression episodes per day. Patients with left bundle branch block at baseline 12-lead ECG were considered unsuitable for ischemia analyses.

Clinical events were acute coronary syndromes, symptomatic tachyarrhythmia, and cardiac arrest. Acute coronary syndromes were defined according to guidelines from the European Society of Cardiology [14].

### Statistics

Continuous variables and counts on Holter recording were adjusted for technically acceptable recording time per day. McNemar test was used to compare proportions before and during treatment, paired *t*-test was employed to compare continuous variables with a Gaussian distribution, and the Wilcoxon signed rank test was used for continuous outcomes with a non-Gaussian distribution. For analyses of repeated measures on continuous outcomes with a non-Gaussian distribution, the Friedman’s test was applied. If the Friedman’s test yielded a significant result, the Wilcoxon pairwise test with Bonferroni correction was applied *post hoc*. All tests were two-sided, and statistical significance was considered at *P* < 0.05. The statistical analyses were performed in IBM SPSS Statistics 22 and SAS version 8.

## Results

**Figure 1:**
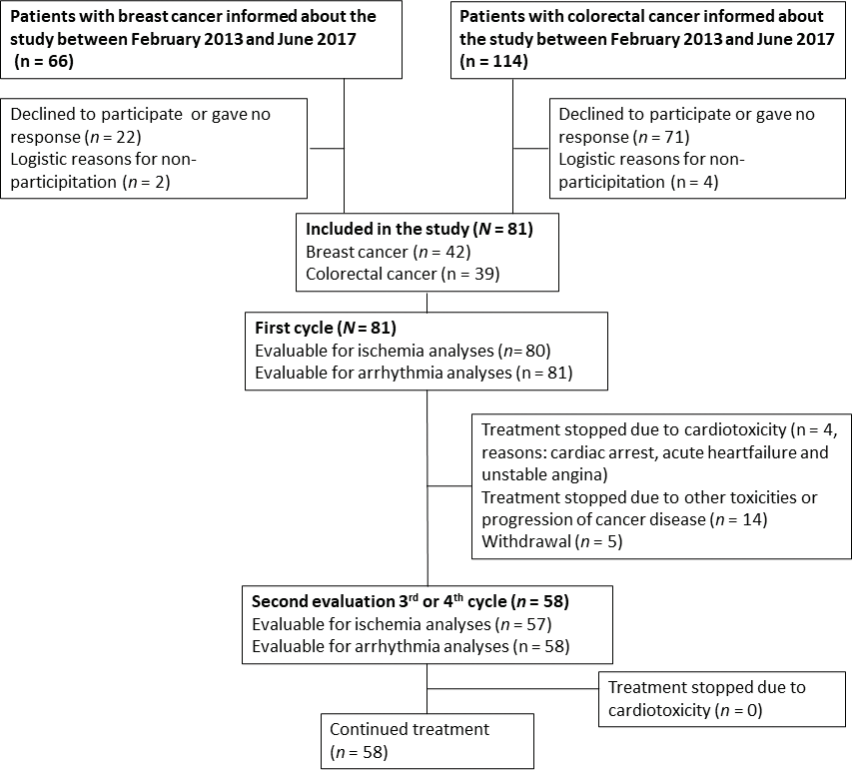
Flow diagram of patient inclusion and study examinations. The diagram shows the inclusion into the study, reasons for exclusion of patients, the number of patients evaluable for ischemia and arrythmia analyses in 1^st^ cycle and 3^rd^/4^th^ cycle, as well as reasons for withdrawal during the study.

A total of 81 patients were included in the study (Figure 1). Of these, 42 had breast cancer, 25 colon cancer, and 14 had rectal cancer (Table 1). While 29 patients (36%) were treated with capecitabine in the (neo)adjuvant setting, 52 (64%) were treated in the metastatic setting. Most of the patients received capecitabine monotherapy (*n* = 67; 83%), while 13 (16%) received capecitabine plus oxaliplatin and 1 patient (1%) received capecitabine plus irinotecan (Table 1).

**Table 1:** Patient characteristics.

	All patients in the study (*N* = 81)	Patients with myocardial ischemia^a^ (*n* = 7)	Patients with clinical events (*n* = 7)	Patients with silent myocardial ischemia (*n* = 4)	Patients without symptoms or myocardial ischemia (*n* = 70)
	*n* (%)	*n* (%)	*n* (%)	*n* (%)	*n* (%)
**Age**					
< 75 years	59 (73)	7 (100)	5 (71)	4 (100)	50 (71)
≤ 75 years	22 (27)	0	2 (29)	0	20 (29)
**Sex**					
Male	23 (28)	0	0	0	23 (33)
Female	58 (72)	7 (100)	7 (100)	4 (100)	47 (67)
**Localisation of cancer**					
Breast	42 (52)	7 (100)	4 (57)	4 (100)	34 (49)
Colon	25 (31)	0	3 (43)	0	22 (31)
Rectum	14 (17)	0	0	0	14 (20)
**Treatment setting**					
Neoadjuvant	2 (3)	0	0	0	2 (3)
Adjuvant	27 (33)	0	3 (43)	0	24 (34)
Metastatic	52 (64)	7 (100)	4 (57)	4 (100)	44 (63)
**Schedule of chemotherapy**					
Capecitabine monotherapy	67 (83)	7 (100)	6 (86)	4 (100)	57 (81)
CAPEOX	13 (16)	0	1 (14.3)	0	12 (17)
CAPIRI	1 (1)	0	0	0	1 (1)
**Performance status**					
0	48 (59)	5 (71)	3 (43)	3 (75)	42 (60)
1	27 (33)	2 (29)	4 (57)	1 (25)	22 (31)
2	3 (4)	0	0	0	3 (4)
**Dose intensity, start**					
90%–100%	57 (70)	7 (100)	5 (71)	4 (100)	48 (69)
70%–80%	23 (28)	0	2 (29)	0	21 (30)
50%	1 (1)	0	0	0	1 (1)
Previous anthracycline	19 (24)	0	1 (14)	0	18 (26)
**Prior chest irradiation**					
Left breast	13 (16)	2 (29)	1 (14)	2 (50)	10 (14)
Right breast	12 (15)	2 (29)	0	2 (50)	10 (14)
Both breasts	3 (4)	0	0	0	3 (4)
Mediastinum	2 (3)	0	0	0	2 (3)
Ischemic heart disease or previous myocardial infarction	3 (4)	0	0	0	3 (4)
Previous Stroke or Transient Cerebral Ischemia	2 (3)	0	0	0	2 (3)
Heart failure	3 (4)	0	0	0	3 (4)
Atrial fibrillation or fluttering	5 (6)	0	0	0	5 (7)
Other heart diseases^b^	1 (1)	0	1 (14)	0	0
Hypertension	38 (47)	2 (29)	3 (43)	1 (25)	34 (49)
Hypercholesterolaemia	26 (32)	0	3 (43)	0	23 (33)
Diabetes Mellitus	7 (9)	0	0	0	7 (10)
**Smoking status**					
Current smoker	9 (11)	1 (14)	2 (29)	0	7 (10)
Former smoker	43 (53)	3 (43)	2 (29)	2 (50)	39 (56)
Never smoked	29 (36)	3 (43)	3 (43)	2 (50)	24 (34)
**Body mass index (BMI)**					
Underweight (BMI < 18.5)	2 (3)	2 (29)	1 (14)	1 (25)	0
Normal (BMI 18.5–24.9)	43 (53)	3 (43)	3 (43)	2 (50)	38 (54)
Overweight (BMI 25.0–29.9)	25 (31)	0	1 (14)	0	24 (34)
Obese (BMI > 29.9)	11 (14)	2 (29)	2 (29)	1 (25)	8 (11)
**Cardiac medications**					
Betablocker	11 (14)	0	1 (14)	0	10 (14)
Calcium channel blocker	10 (12)	0	0	0	10 (14)
Digoxin	4 (5)	0	0	0	4 (6)
**NYHA class at baseline**					
1–2	77 (95)	7 (100)	7 (100)	4 (100)	46 (66)
3	4 (5)	0	0	0	4 (6)
** (*n* = 52)**					
< 50%	11 (14)	0	0	0	11 (16)
Low eGFR (< 60 mL/min/1.73m^2^)	4 (5)	0	0	0	4 (6)
**Anaemia^c^**	25 (31)	0	0	0	25 (36)
1	20 (25)	0	0	0	20 (25)
2	6 (7)	0	0	0	6 (7)

NYHA: New York Heart Association; LVEF: left ventricular ejection fraction; SD: standard deviation.

a Primary endpoint, myocardial ischemia, defined as patients with acute coronary syndromes and/or myocardial ischemia on 12-lead ECG or Holter recording. This group includes three patients with clinical events and all four patients with silent ischemia.

b Aortic valve stenosis.

c CTCAE version 5.0: Grade 1, <Lower Limit of Normal – 6.2 mmol/L; grade 2, <6.2 mmol/L – 4.9 mmol/L. No patients had grade 3 or 4 anaemia. Lower Limit of Normal for the assay was 7.3 mmol/L for women and 8.3 mmol/L for men. Patients with haemoglobin below 5.3 mmol/L received blood transfusion before treatment start.

Holter recording was obtained in all patients during the 1^st^ cycle of treatment. One patient was excluded from ischemia analyses due to left bundle branch block (Figure 1). A total of 23 patients did not receive 3^rd^ or 4^th^ cycle of capecitabine due to adverse events, progression of cancer disease, or due to withdrawal of consent (Figure 1). In all, 58 patients were available for Holter recording before and during 3^rd^ or 4^th^ cycle, and 57 were evaluable for ischemia analyses (one left bundle branch block). Median recording time before and during treatment is shown in Supplementary Table S4.

### Myocardial ischemia

#### Day-to-day variation in ischemic episodes

A considerable day-to-day variation in the amount of myocardial ischemia was seen during treatment (Supplementary Figure S2A and B).

#### Myocardial ischemia before and during capecitabine treatment

**Figure 2:**
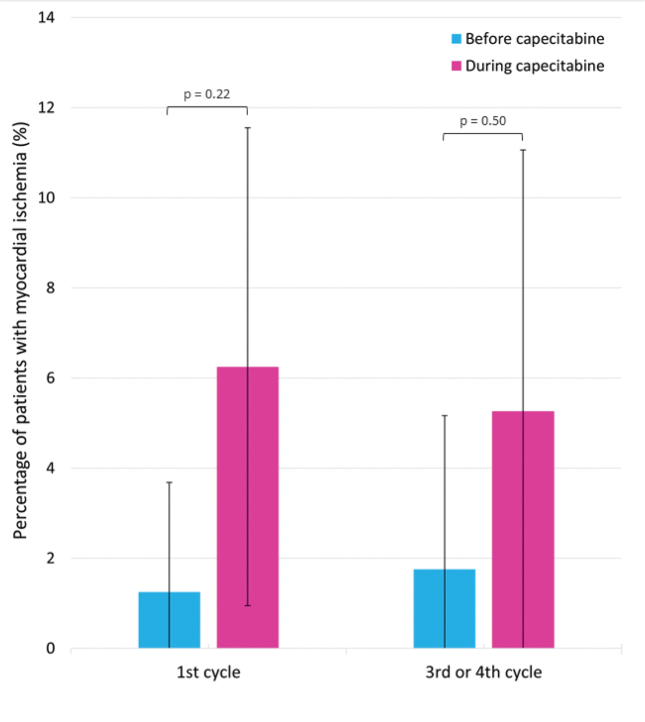
Myocardial ischemia on Holter recording before and during capecitabine treatment. The figure shows the percentage of patients with myocardial ischemia on Holter recording before and during 1^st^ cycle and 3^rd^/4^th^ cycle. The error bars illustrate the 95% confidence intervals. The p-values above the bars represent the p-values from the McNemar test comparing the number of patients with myocardial ischemia before and during treatment with capecitabine.

Myocardial ischemia was seen on Holter recording in six patients (7%) during either the 1^st^ cycle or the 3^rd^/4^th^ cycle of treatment (Supplementary Tables S5). Of these, four patients (5% of all patients) had silent myocardial ischemia, while two (2%) had clinical events. Among the patients with myocardial ischemia, five (6%) had myocardial ischemia during 1^st^ cycle and three (5%) during 3^rd^/4^th^ cycle (1 patient had myocardial ischemia during both 1^st^ and 3^rd^/4^th^ cycle) (Figure 2). Although numerically more patients had myocardial ischemia on Holter recording during treatment compared to before treatment start, the difference was not statistically significant, neither in 1^st^ cycle (*p* = 0.22) nor in 3^rd^/4^th^ cycle (*p* = 0.50). Similarly, there were no differences before and during treatment in the ischemic burden per day (1^st^ cycle: *p* = 0.23, 3^rd^/4^th^ cycle: *p* = 0.11), the number of ST-elevation episodes per day (1^st^ cycle: *p* = 0.29, 3^rd^/4^th^ cycle: *p* = 1.0), the number of ST depression episodes per day (1^st^ cycle: *p* = 0.18, 3^rd^/4^th^ cycle: *p* = 0.11). and the total duration of ischemic episodes per day (1^st^ cycle: *p* = 0.23, 3^rd^/4^th^ cycle: *p* = 0.11) (Supplementary Table S7).

### Clinical events

Fifteen patients reported possible cardiac symptoms during treatment (14 in the 1^st^ cycle, 1 in the 4^th^ cycle) and in seven patients (9%) the symptoms were interpreted as cardiotoxicity (Supplementary Figure S3). Among them, four experienced unstable angina without myocardial ischemia (5%), one exhibited unstable angina accompanied by myocardial ischemia on Holter recording (1%), one developed dyspnoea, myocardial ischemia on Holter recording and reduced ejection fraction on echocardiography (1%), and one patient sustained sudden cardiac arrest after Holter removal (1%) (Supplementary Table S5). None of the patients with clinical events had cardiac symptoms or myocardial ischemia before treatment start. Five patients presented with symptoms in 1^st^ cycle while two had debut of symptoms in later cycles. Treatment with capecitabine was stopped due to cardiotoxicity in four patients, while the dose was reduced in 1.

### Combined outcome of clinical events and myocardial ischemia

The incidence of the combined outcome of myocardial ischemia and clinical events during treatment with capecitabine was 14% (*n* = 11). Before 1^st^ cycle only one patient (1%) had myocardial ischemia, while seven patients (9%) experienced myocardial ischemia or clinical events during 1^st^ cycle (*p* = 0.070). During 3^rd^/4^th^ cycle five patients (9%) had had myocardial ischemia or clinical events compared to one patient before 3^rd^ or 4^th^ cycle (2%) (*p* = 0.13).

### Biomarkers

Elevations in cTnI above the cut-off level were observed in six patients during the study. Among those were one patient with persistently high troponin throughout the treatment, three with fluctuating troponin levels, and two with rises in cTnI above the cut-off level during 1^st^ cycle of treatment (Supplementary Table S8). One of the two patients with a rise in cTnI was the patient with sudden cardiac arrest, while the other patient with rise in cTnI had neither symptoms nor myocardial ischemia. Among those with fluctuations in cTnI were one with angina pectoris.

Increases in cTnI plasma concentrations larger than the assay variation but below the cut-off level for myocardial infarction were observed in four patients (5%) during 1^st^ cycle and in three patients (5%) during 3^rd^/4^th^ cycle. Among those was one patient with clinical event and myocardial ischemia on Holter recording.

**Figure 3:**
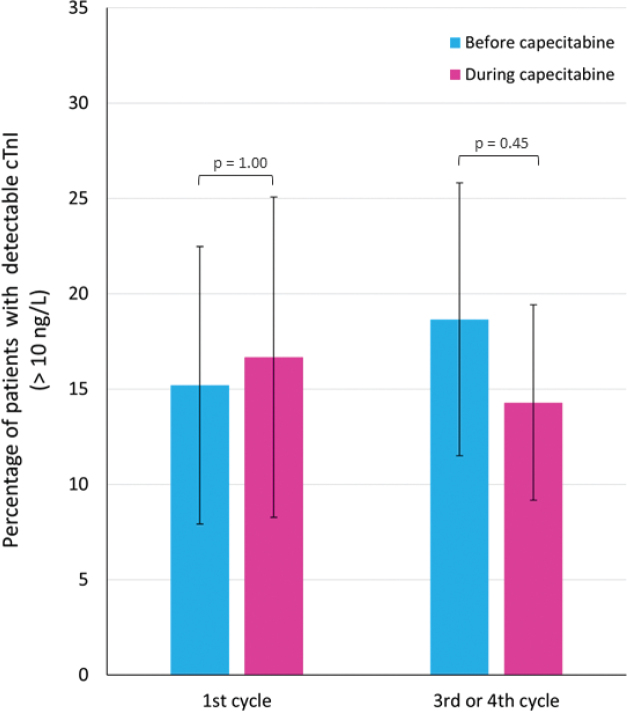
Plasma cardiac troponin I (cTnI) levels before and during capecitabine treatment. The figure illustrates the percentage of patients with detectable plasma cTnI levels (lower limit of detection: 10 ng/L) before and during treatment with capecitabine in 1^st^ cycle and 3^rd^/4^th^ cycle, respectively. The error bars illustrate the 95% confidence intervals. The p-values above the bars represent the p-values from the McNemar test comparing the number of patients with detectable cTnI values before and during treatment with capecitabine.

There was no difference in plasma cTnI levels before and during treatment in 1^st^ cycle (*p* = 0.92) or 3^rd^/4^th^ cycle (*p* = 0.48) (Supplementary Figure S4). Furthermore, there were no differences in the number of patients with plasma troponin levels above the lower limit of detection before and during capecitabine (Figure 3).

**Figure 4:**
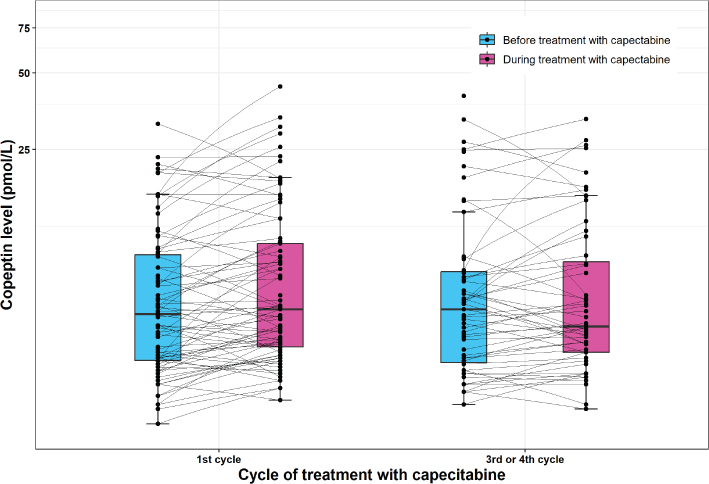
Plasma copeptin levels before and during treatment with capecitabine. The figure shows the plasma copeptin levels before and during treatment with capecitabine in 1^st^ cycle and 3^rd^ or 4^th^ cycle, respectively. The black line in the middle of the boxes represents the median, the lower boundary of the boxes represents the 25% quartile, the upper boundary of the boxes represents the 75% quartile, and the whiskers represents 1.5 times the 25% and 75% quartiles, respectively. In first cycle, the median plasma copeptin level were higher during treatment compared to before (Wilcoxon signed rank test, *p* = 0.004), while there was difference in copeptin levels before and during the 3^rd^/4^th^ cycle (Wilcoxon signed rank test, *p* = 0.15). Normal range for copeptin in healthy volunteers is 1.70–11.25 pmol/L (2.5 and 97.5 percentile, respectively).

The median plasma copeptin level increased from 5.6 pmol/l (range: 2.1–31.5 pmol/l) before the 1^st^ cycle to 5.9 pmol/l (range: 2.6–44.2 pmol/l) during the 1^st^ cycle (Wilcoxon signed rank test, *p* = 0.004) (Figure 4). There was no significant difference in copeptin levels before (median 5.9, range 2.5–40.6 pmol/l) and during (median 5.1, range 2.4–32.9 pmol/l) the 3^rd^/4^th^ cycle (*p* = 0.15). Copeptin values above the suggested cut-off for myocardial infarction (>10 pmol/L) were observed in 26 patients (32%). There were no differences in the number of patients with elevated copeptin before and during treatment in 1^st^ cycle (*p* = 0.45) or 3^rd^/4^th^ cycle (*p* = 0.22) (Supplementary Table S9). Among those with elevated copeptin, a rise was observed in 13 patients during 1^st^ cycle and in nine patients during 3^rd^/4^th^ cycle. None of the patients with myocardial ischemia on Holter recording during treatment had copeptin levels >10 pmol. Furthermore, only one patient with copeptin >10 pmol/l had elevated troponin above the cut-off for myocardial infarction; however, this patient did not exhibit symptoms or myocardial ischemia.

### Non-sustained ventricular tachycardia

During 1^st^ cycle of treatment, more patients had episodes with non-sustained ventricular tachycardia compared to before treatment (*p* = 0.021) (Supplementary Table S10). This was also seen across the days of treatment in 1^st^ cycle (*p* = 0.044, not significant after correction for multiple tests with Bonferroni correction, Supplementary Table S11). In total, 13 patients had new onset non-sustained ventricular tachycardia during 1^st^ cycle.

In the 3^rd^/4^th^ cycle, new onset non-sustained ventricular tachycardia was present in nine patients during capecitabine and there was no difference in the number of patients with episodes with non-sustained ventricular tachycardia during versus before treatment (*p* = 0.065).

No patients with myocardial ischemia or clinical events had episodes of non-sustained ventricular tachycardia, but the patient with cardia arrest had sustained ventricular tachycardia before defibrillation.

### QTc on 12-lead ECG

There was no difference in the geometric mean of the QTc interval before and during treatment neither in 1^st^ cycle (417 ms, 95% confidence interval [CI]: 410–424 ms vs. 412 ms, 95% CI: 406–420 ms, *p* = 0.17) nor in 3^rd^/4^th^ cycle (416 ms, 95% CI: 408–423 ms vs. 414 ms, 95% CI: 407–421 ms, *p* = 0.60).

## Discussion

This prospective study of capecitabine-induced cardiotoxicity showed an incidence of myocardial ischemia of 7% on Holter recording and an incidence of clinical events of 9%. We could not demonstrate an increase in myocardial ischemia or in the combined outcome of clinical events and myocardial ischemia during treatment with capecitabine compared to before. Elevations in plasma cTnI levels were rare, while an increase in median copeptin levels and an increase in the frequency of non-sustained ventricular tachycardia episodes were found during 1^st^ cycle.

The incidence of symptomatic cardiotoxicity of 9% in the present study is in line with prior studies reporting incidences between 1.9% to 35.0% [6, 26], with most prospective studies reporting incidences of 3–7% [8, 10, 27, 28]. Furthermore, the incidence of symptomatic cardiotoxicity of 9% in the present study is not significantly different from the incidence of 6% found in our previous study of 5-FU (DeGramont regimen) cardiotoxicity with similar design (9% vs. 6%, 95% CI −5% to 11%, *p* = 0.403) [11]. On the other hand, the incidence of myocardial ischemia of 7% during capecitabine in the present study is significantly lower than the incidence of myocardial ischemia of 19% during 5-FU treatment in our previous study (difference 12 percent point, 95% CI of the difference 2% – 21%, *p* = 0.029) [11].

Few prior studies have performed Holter recording during capecitabine treatment, but a prospective study evaluated myocardial ischemia and ventricular arrhythmias during exercise stress-test in patients treated with capecitabine [10]. In this study, 3% had myocardial ischemia at rest, while 13.5% had a positive exercise stress-test (myocardial ischemia or ventricular arrhythmias) during treatment [10]. The authors reported a total incidence of cardiotoxicity of 16.7% [10], which is slightly higher than the combined incidence of myocardial ischemia and clinical events of 14.0% in the present study. The main reasons for the different findings may be different methods used (exercise stress test vs. Holter recording), the timing of the examinations (first 4–7 days of treatment in our study; day 10–38 in the study by Lestuzzi et al. [10]), and the criteria for cardiotoxicity.

We did not observe a statistically significant increase in myocardial ischemia during treatment compared to the pre-treatment period. This differs from our previous study of 5-FU, in which the number of patients experiencing myocardial ischemia, the ischemic burden, the total duration of myocardial ischemia, and the number of ST-depression episodes rose during treatment with 5-FU (administered as the DeGramont regimen) [11]. The lack of statistical significance in the current study could be due to smaller sample size and the lower occurrence of myocardial ischemia during capecitabine compared to 5-FU. The lower occurrence of myocardial ischemia during capecitabine may be because of lower plasma concentrations of 5-FU following capecitabine compared to intravenous 5-FU administration [29, 30]. Capecitabine undergoes a 3-step conversion process to 5-FU, with the final step being catalysed by thymidine phosphorylase – an enzyme with higher activity in many types of tumour tissues [31]. Consequently, the concentration of 5-FU following capecitabine is higher in tumour tissues, but lower in plasma compared to intravenous 5-FU [29, 30].

Silent myocardial ischemia occurred more frequently than symptomatic myocardial ischemia. The significance of silent myocardial ischemia is unclear, but in patients with coronary heart disease, as well as in middle-aged and elderly individuals without a heart disease diagnosis, silent myocardial ischemia is associated with an increased risk of myocardial infarction and sudden death [32, 33].

In concordance with prior studies of fluoropyrimidine cardiotoxicity [16–19], elevations in cTnI above the diagnostic cut-off for myocardial infarction were rare and median plasma cTnI levels remained unchanged during treatment. Yet, a subset of patients had fluctuations in plasma cTnI higher than the assay variation. For most patients, the fluctuations were not related to myocardial ischemia on Holter recording. It is well-known that elevated cardiac troponin levels can result from various cardiac and non-cardiac diseases, acute illnesses, and even false-positive results due to factors that affect assay performance [34, 35]. Thus, there may be other reasons for the observed fluctuations in plasma cTnI levels in some patients.

We found a slight, yet statistically significant rise in copeptin levels during 1^st^ cycle of capecitabine, but not in the later cycles. This is in line with findings from our study of 5-FU cardiotoxicity [11], but the clinical significance is unknown. In the present study, 21% of the patients had copeptin elevations before treatment and copeptin elevations were mainly seen in patients without myocardial ischemia and without elevations in cTnI, questioning its relation to cardiotoxicity.

We observed an increase in episodes of non-sustained ventricular tachycardia during 1^st^ cycle of treatment. Prior Holter studies in patients receiving 5-FU have demonstrated conflicting results with increased number and complexity of ventricular premature complexes during 5-FU in one study [36] and no differences in the incidence of ventricular arrhythmias before and during treatment in two studies [11, 37]. Recently, a large study based on registry data, found increased incidence of cardiac arrhythmias in patients with cancer receiving fluoropyrimidines compared to patients with cancer not receiving fluoropyrimidines [38]. However, the study pooled ventricular and supraventricular arrhythmias, limiting the interpretation of the findings.

The clinical significance of non-sustained ventricular tachycardia episodes in relation to the risk of severe cardiotoxicity, for example, cardiac arrest, is unclear. Case reports of patients with fluoropyrimidine cardiotoxicity have demonstrated coronary vasospasm [39, 40] suggesting that myocardial ischemia is the major reason for cardiotoxicity and in our 5-FU study, cardiac arrest was preceded by major ST elevations in a patient further supporting myocardial ischemia as the major reason for severe cardiotoxicity [11]. In the present study, non-sustained ventricular tachycardia was observed in patients without myocardial ischemia indicating that ventricular arrhythmia is not only seen secondary to myocardial ischemia but may occur as a separate clinical event.

Fluoropyrimidine cardiotoxicity covers a spectrum of cardiac events from mild chest pain to cardiac arrest [6, 26]. Clinicians are often challenged when deciding whether a patient with cardiotoxicity should continue treatment or hold treatment, since cardiotoxicity is often recurrent [7, 41] while discontinuation of treatment may withhold the patient a beneficial treatment. In this context, it is challenging that the results from the different modalities for example Holter recording, biomarkers, and ECGs do not always correlate with each other and with the symptoms of the patient. Silent myocardial ischemia may represent early signs of cardiotoxicity but till date there are no reliable early markers of fluoropyrimidine cardiotoxicity.

Future studies could use rubidium positron emission tomography (PET) or cardiac magnetic resonance imaging (MRI) to further elucidate the effects of fluoropyrimidines on the heart. Both modalities are non-invasive and can address myocardial perfusion [42, 43] which may be compromised during fluoropyrimidine cardiotoxicity leading to myocardial ischemia. Furthermore, cardiac MRI can show detailed pictures of the anatomy of the heart and give precise measures of ejection fraction [42].

### Strengths and limitations

Our study might have reached more clear results by including more patients; however, this is among the first Holter studies in patients treated with capecitabine. We chose Holter recording because it is an inexpensive and non-invasive method to detect myocardial ischemia during daily life. A standard Holter recorder can record two leads for up to 7 consecutive days. Thus, it was not possible to monitor the patients during all 14 days of capecitabine. Even though, capecitabine cardiotoxicity mostly occurs during the first week of treatment [44], cardiotoxicity and myocardial ischemia may occur later in the cycles after removal of the Holter recorder.

Only two leads were available on Holter recording, limiting the detection of myocardial ischemia to the area of the myocardium covered by those two leads [11]. Nevertheless, lead CM5 has been documented to exhibit a sensitivity of 89% in detecting myocardial ischemia, compared to a 12-lead ECG [45].

Factors such as positional movements, left ventricular strain patterns, and ST elevations due to high blood pressure may influence the ST-segment on Holter recording complicating the interpretation of ST-segment. To address the influence of positional movements, we obtained 1-min recordings in the supine, sitting, and standing positions in a subgroup of patients (Supplementary material p. 4), and found no significant ST-deviations during positional movements. Also, the prevalence of myocardial ischemia before treatment start was low (1.3%) in our study compared to incidences in population-based studies of middle-aged to elderly subjects (6% – 35%) [33, 46] suggesting that we did not overestimate the incidence of myocardial ischemia.

Finally, we reviewed the Holter recordings for ischemic episodes and ventricular tachyarrhythmias on the day of Holter recorder removal. This might in some patients have led to discontinuation of treatment before major events occurred.

## Conclusions

Capecitabine was associated with an incidence of clinical cardiotoxicity of 9% and an incidence of myocardial ischemia of 7% on Holter recording. The frequency of episodes with non-sustained ventricular tachycardia and the median plasma copeptin levels rose during 1^st^ cycle of treatment, while increases in cTnI levels were rare. The total incidence of clinical cardiotoxicity and myocardial ischemia of 14% highlights the importance of this side effect. However, symptoms, changes on Holter monitoring, and increases in cardiac biomarkers do not always correlate, complicating the interpretation of these findings.

Future research should aim to clarify the significance of asymptomatic electrocardiographic changes and identify factors that predispose to severe fluoropyrimidine-induced cardiotoxicity. In addition, gaining a deeper understanding of the pathophysiology is crucial, and studies utilising cardiac MRI or rubidium PET could provide valuable information.

## Supplementary Material



## Data Availability

Danish legislation prohibits sharing patient data. Researchers need to apply the Danish Health Data Authority to have access to the underlying person-level data. For interest in collaborative studies, please contact the corresponding author.
