# Supplementary material for: Incidence of myocardial ischemia during treatment with capecitabine: a cohort study with Holter recording and cardiac biomarkers
Source: Acta Oncol. 2025 Jul 2;64:43089. doi: 10.2340/1651-226X.2025.43089 (PMC12239133; doi:10.2340/1651-226X.2025.43089)
Supplement: Supplementary file 1 [file AO-64-43089-s1.pdf]

Supplementary material has been published as submitted. It has not been copyedited, or typeset by Acta Oncologica

SUPPLEMENTARY MATERIAL

Incidence of myocardial ischemia during treatment with capecitabine: A cohort study with Holter recording and cardiac biomarkers

Content

Supplementary Material: Methods ... 2

Table S1: Treatment regimens ... 2

Table S2: Definition of cardiovascular risk factors ... 3

Figure S1: Placement of Holter electrodes on the chest wall ... 4

Influence of positional movements on the ST-segment ... 4

Table S3: Endpoint definitions and methodological details ... 5

Study/sample size calculation ... 6

Supplementary Material: Results ... 7

Table S4: Median recording time ... 7

Day-to-day variation of ischemic episodes before capecitabine treatment ... 8

Day-to day variation in ischemia during capecitabine treatment ... 8

Figure S2 A and B: Ischemic burden per day of recording (log scale) for patients with myocardial ischemia during 1st cycle and 3rd or 4th cycle, respectively ... 8

Table S5: Details concerning patients with cardiac symptoms and myocardial ischemia ... 9

Figure S3: Diagnostic work-up for patients with symptoms during capecitabine treatment ... 11

Table S8: Cardiac troponin I levels in patients with cardiac troponin I above the cut-off level of 40 ng/L during the study period or increases in cardiac Troponin I levels larger than the assay variation but below cut-off level ... 12

Figure S4: Median plasma cardiac troponin I levels before and during treatment with capecitabine ... 13

Table S9: Number of patients with elevated copeptin before and during treatment ... 14

Table S10: Number of patients with episodes of non-sustained ventricular tachycardia before and during treatment with capecitabine ... 15

Table S11: Non-sustained ventricular tachycardia according to the different days in cycle 1: P-values from Wilcoxon signed rank test without and with Bonferroni correction ... 16

Discussion of the differences in study design of this study compared to our previous study of 5-FU ... 17

Alternative treatment options and S-1 ... 17

References to the supplementary material ... 19

Supplementary Material: Methods

Table S1: Treatment regimens

Treatment regimen	Administration
Capecitabine monotherapy	Capecitabine (2000 mg/m^2) in 14 days followed by 7 days off *
CAPOX	Capecitabine (2000 mg/m^2) in 14 days followed by 7 days off + oxaliplatin (130 mg/m^2) **
CAPIRI	Capecitabine (1600 mg/m^2) in 14 days followed by 7 days off + irinotecan (200 mg/m^2) **
Neoadjuvant CAPOX followed by radiation and concomitant capecitabine	Capecitabine (2000 mg/m^2) in 14 days followed by 7 days off + oxaliplatin (130 mg/m^2), 3 cycles<br>Chemoradiation with capecitabine 1800 mg/m^2 per day, continuously until end of radiation therapy **

* Antiemetics: Domperidone 10 mg as needed, up to three doses per day

** Antiemetics: Prednisolone 50 mg day 1–3, ondansetron 16/24 mg day 1 and domperidone 10 mg as needed, up to three doses per day

Table S2: Definition of cardiovascular risk factors

Hypertension	A medical history or self-reported diagnosis of hypertension or current intake of antihypertensive medications
Hypercholesterolemia	A medical history or self-reported diagnosis of hypercholesterolemia or current intake of cholesterol lowering medications and/or a non-fasting total cholesterol > 5.0 mmol/L [1].
Diabetes	A medical history or self-reported diagnosis of diabetes or current intake of anti-diabetic medications and/or a fraction of glycosylated hemoglobin (HbA1c) > 48 mmol/mol [2].
Smoking	Self-reported smoking habits<br>Categorized as current smoker, former smoker or never smoked
Body mass index days	Calculated from height and weight<br>Categorized according to WHO's classification in:<br>Underweight (< 18.5 kg/m^2)<br>Normal (18.5–24.9 kg/m^2)<br>Overweight (25.0–29.9 kg/m^2)<br>Obese (> 29.9 kg/m^2)

Figure S1: Placement of Holter electrodes on the chest wall

Del Mar Reynolds Medical

PROCEDURES USING 3, 4 AND 6 ELECTRODE PATIENT CABLES

Three electrode patient cable

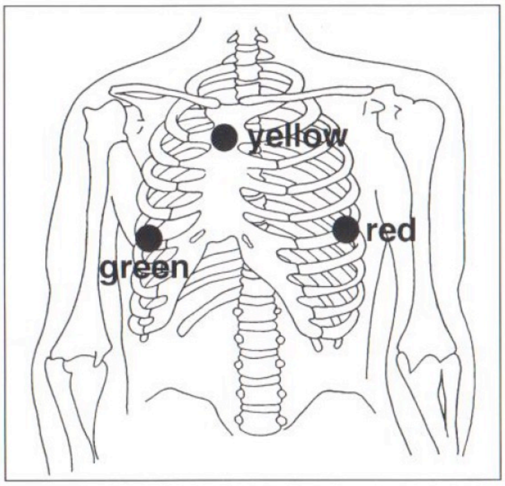

Yellow CH1 - CH3	On the right sternal border at the level of the 2nd rib.
Red CH1 + CH2 +	In the left anterior axiliary line and on the 6th rib.
Green CH2 - CH3 +	In the right anterior axiliary line and on the 6th rib.

Red/Yellow = CH1 = CM5 Red/Green=CH2 = CC5 Green/Yellow = CH3 = CM5R

Procedures using 3 electrodes	90 Mb CF card	ECG Channels
Standard Mode (Start...)	48 hours	1, 2 and 3
Extended Mode (Start Week...)	7 days	1 and 2

Holter recording were performed for 1–3 days before treatment start and up to 4-6 days during treatment with capecitabine.

Instructions of the patients

- During recording the patients were allowed only to remove the Holter monitor briefly during showering and they received detailed instructions together with an illustration on how to apply and replace the electrodes and cables correctly.
- No instructions about physical activity during the study period were given, but the patients were instructed to record their activities in a diary.

Influence of positional movements on the ST-segment

The influence of positional movements on the ST segment were examined in a subgroup of 25 consecutive patients by obtaining 1-minute recordings in the supine, sitting and standing positions No significant ST-deviations were observed during these positional movements.

Table S3: Endpoint definitions and methodological details

Clinical events	Including acute coronary syndromes, symptomatic tachyarrhythmias and cardiac arrest.<br>Acute coronary syndromes and myocardial infarction were defined according to current guidelines from the European Society of Cardiology [3].
Myocardial ischemia on Holter recording	ST elevation of ≥ 1 mV measured in the J-point lasting at least 1 minute or downsloped or horizontal ST depression of ≥ 1 mV measured 60 ms after the J-point lasting at least one minute.<br>An interval of ≥ 1 minute of recording with no ST deviations should be present before a new discrete episode was counted.<br>The PR-segment was used as reference point, but we corrected for baseline ST-abnormalities [4].
Myocardial ischemia on 12-lead ECG	Significant ST elevation or significant ST depression in at least two adjacent leads or negative T-waves of ≥ 0.1 mV in two adjacent leads with prominent R or R/S > 1.<br>ST elevation was measured in the J-point and considered significant if ≥ 0.25 mV in V2-V3 for men < 40 years old, ≥ 0.20 mV in V2-V3 for men > 40 years old, ≥ 0.15 mV in V2-V3 for women and ≥ 0.10 mV in all other leads.<br>ST depression was measured 60 ms after the J-point and horizontal or downsloped depressions of ≥ 0.05 mV in at least two adjacent leads were considered significant [3].
Cardiac troponin I, elevations and fluctuations	Assay: ADVIA Centaur TnI-Ultra assay, Siemens Healthcare Diagnostics Inc (upper 99th percentile cut-off: 40 ng/L, coefficient of variation at the 99th percentile: 10%, calibration traceable to NIST SRM 2921) [5].<br>Elevations: Defined as values above the upper 99th percentile cut-off of 40 ng/L [5].<br>Fluctuations: Increases in cardiac troponin I plasma concentrations larger than the assay variation but below the 99th percentile. According to the assay specifications an increase in troponin of 44.4% is considered clinically significant.
Copeptin	Assay: Thermo Scientific™ BRAMS™ Copeptin pro-vasopressin immunofluorescent assay, Thermo Fischer Diagnostics (lower detection limit: 0.69 pmol/L, intra-assay coefficient of variation < 15%, inter-assay coefficient of variation < 18%, 2.5 and 97.5 percentiles 1.7 and 11.25 pmol/L, respectively) [6].<br>Copeptin was analyzed as a continuous variable.<br>Secondly, the number of patients with co-peptin levels above the suggested cut-off for myocardial infarction (10 pmol/L) is given [7].
Ventricular tachyarrhythmia (both 12 lead ECG and Holter)	≥ 3 complexes with a QRS interval > 120 ms and ≥ 100 beats per minute [8].<br>Non-sustained ventricular tachycardia: duration < 30 seconds, patient hemodynamic stable.<br>Sustained ventricular tachycardia: duration > 30 seconds, patient hemodynamic instable
QTc	QT was measured on resting 12-lead ECG and corrected by use of Bazett's formula [9].

Study/sample size calculation

Sample size was estimated from the expected proportion of patients with cardiotoxicity using the following equation:

$$n \geq \left(\frac{z}{m}\right)^2 \times \hat{p}(1-\hat{p})$$

Where m is the margin of error and $\hat{p}$ is the expected proportion. z = 1.96 for 95% confidence intervals.

For capecitabine the expected proportion of patients with myocardial ischemia was set to 10% and a margin of error og 7 was chosen/accepted:

$n \geq (1{,}96/0{,}07)^2 \times 0{,}1 \times (1-0{,}1) = 71$ patients

Supplementary Material: Results

Table S4: Median recording time

	Before capecitabine (hours)	During capecitabine (hours)
First cycle	34.6 (range 7.8–128.1)	132.5 (range 47.0–160.1)
Second recording (3rd or 4th cycle)	30.3 (range 7.7–78.8)	134.2 (range 24.8–162.8)

Day-to-day variation of ischemic episodes before capecitabine treatment

Only one patient had myocardial ischemia on Holter recording before first cycle, and one had before 3rd or 4th cycle. Both patients had only one day of monitoring before treatment start and therefore it was not possible to estimate the day-to-day variation in ischemia before treatment start.

Day-to day variation in ischemia during capecitabine treatment

The day-to-day variation in ischemic burden during capecitabine treatment is shown graphically for patients with myocardial ischemia in Figure A3a and b.

Figure S2 A and B: Ischemic burden per day of recording (log scale) for patients with myocardial ischemia during 1st cycle and 3rd or 4th cycle, respectively

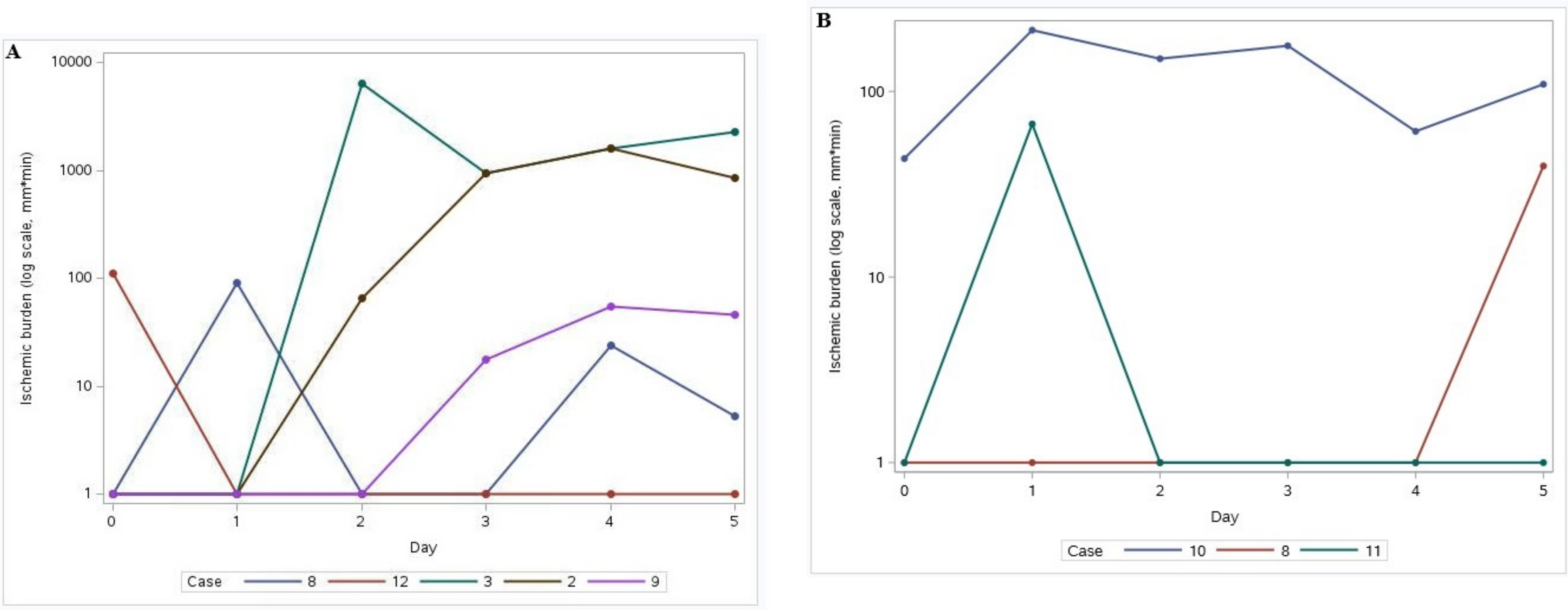

Figure S2 A and B shows the ischemic burden on a log scale (Y-axis) per day (X-axis) of Holter recording for cases with myocardial ischemia in 1st cycle (A) and 3rd or 4th cycle (B), respectively. The case number refer to the case numbers in Table S5. The ischemic burden is calculated by multiplication of the amplitude and the duration of ST-deviations on Holter recording, in the channel with most pronounced ischemia. Values of 0 is set to 1 since it is not possible to log-transformed values of 0.

Table S5: Details concerning patients with cardiac symptoms and myocardial ischemia

Case no.	Treatment	Dose intensity of capecita-bine	Cycle of onset (day)	Symptoms	Type of event	CTC AE grade (version 5)	Ischemia before 1st cycle	Ischemia during 1st cycle	Ischemia before 3rd/4th cycle	Ischemia during 3rd/4th cycle	ECG changes on 12-lead ECG	Elevated cTnI or CK-MB	Other findings	Initiated cardiac therapy	Retreat-ment with capecita-bine, dose intensity	Symptoms at retreat-ment
**Patients with cardiac symptoms**																
1	Met. Capecitabine	100%	1 (9)	Suddenly unconscious	Cardiac arrest	4	No	No, not monitored during event	-	-	ST↓ V2-V5, AFLI	cTnI ↑ CK-MB ↑	ECHO: LVEF 45%, otherwise normal<br>CT of chest: ia<br>CAG: no stenoses	CPR, defibrillation, ROSC, therapeutic hypothermia, ASA, clopidogrel	No	-
2	Met. Capecitabine	100%	1 (10)	Dyspnea	Acute heart failure and unstable angina	3	No	ST ↑ day 3-5	-	-	No	No, but fluctuation in cTnI below threshold	NT-proBNP ↑↑<br>ECHO: LVEF 45%, anterolateral hypokinesia<br>Cardiac monitoring: ST ↑ in inferior and anterolateral leads, negative T waves	No, spontaneous recovery after capecitabine withdrawal	No	-
3	Met. Capecitabine	75%	1 (1)	Chest pain, dyspnea	Unstable angina	3	No	ST ↑ day 2-5	-	-	ST ↑ in I, II, III and aVF	No	NT-proBNP ↑<br>Echo: Normal	ASA single dose, calcium antagonist	No	-
4	Adj. CAPOX	100%	3 (4)	Chest pain	Unstable angina	3	No	No	No	No	No	No	No	Calcium antagonist	Yes, 50%	No
5	Adj. capecitabine	75%	1 (3)	Chest pain	Unstable angina	2	No	No	-	-	No	No, but fluctuating cTnI under cut-off level	NT-proBNP ↑ (before and during)	No	No	-
6	Met. Capecitabine	100%	1 (1+2)	Chest pain	Unstable angina	1	No	No	No	No	No	No	No	No	Yes, 100%	No
7	Adj. capecitabine	75%	4 (1)	Chest pain	Unstable angina	1	No	No	No	No	No	No	No	No	Yes, 100%	No
**Patients with silent myocardial ischemia**																
8	Met. cape	100%	1 (1)	None	Silent MI		No	ST ↓ (day 1+4+5)	No	ST ↓ (day 5+6)	No	No	No	No	Yes, 100%	No
9	Met. cape	100%	1 (3)	None	Silent MI		No	ST ↓ (day 3-5), negative T-waves	-	-	No	No	No	No	No	-
10	Met. cape	100%	3 (1)	None	Silent MI		No	No	ST ↓	ST ↓ (day 1-7)	No	No	No	No	Yes, 100%	No
11	Met. cape	100%	3 (1)	None	Silent MI		No	No	No	ST ↓ (day 1)	No	No	No	No	Yes, 100%	No
12	Met. cape	100%	-	None	Silent MI	-	ST ↑	No	No	No	No	No	No	No	Yes, 100%	No

Adj., adjuvant; Met., metastatic; ECG, electrocardiogram; STEMI, ST-elevation myocardial infarction; MI, myocardial ischemia; ST ↓, ST depression; ST ↑, ST elevation; NSVT, non-sustained ventricular tachycardia; VF, ventricular fibrillation; cTnI, cardiac troponin I; CK-MB, creatine kinase MB; ECHO, echocardiography; LVEF, left ventricular ejection fraction; CAG, coronary angiography; CPR, cardiopulmonary resuscitation; ROSC, return of spontaneous circulation; ASA, acetylsalicylic acid; NTG, nitroglycerin

Table S7: The mean number and mean duration of ischemic episodes and the mean total ischemic burden (per patient per 24 hours)

	Before 1st cycle (n = 80)	During 1st cycle (n = 80)	Before 3rd or 4th cycle (n = 57)	During 3rd or 4th cycle (n = 57)
Total ischemic burden[a] (mm*min)	1.4 (SD 12.3)	36.3 (SD 256.7)	0.8 (SD 5.8)	2.6 (SD 16.8)
Total duration of episodes with ST-depression[a] (min)	0	0.3 (SD 2.1)	0.3 (SD 2.2)	1.4 (SD 8.3)
Total duration of episodes with ST-elevation[a] (min)	0.7 (SD 6.7)	14.7 (SD 105.1)	0	0
Number of episodes with ST-depression[a]	0	0.02 (SD 0.11)	0.02 (SD 0.15)	0.12 (SD 0.66)
Number of episodes with ST-elevation[a]	0.02 (SD 0.16)	0.45 (SD 3.1)	0	0
Number of patients with both symptomatic and silent ischemic episodes	0	2	0	0

[a]per patient per 24 hours; SD = standard deviation

Figure S3: Diagnostic work-up for patients with symptoms during capecitabine treatment

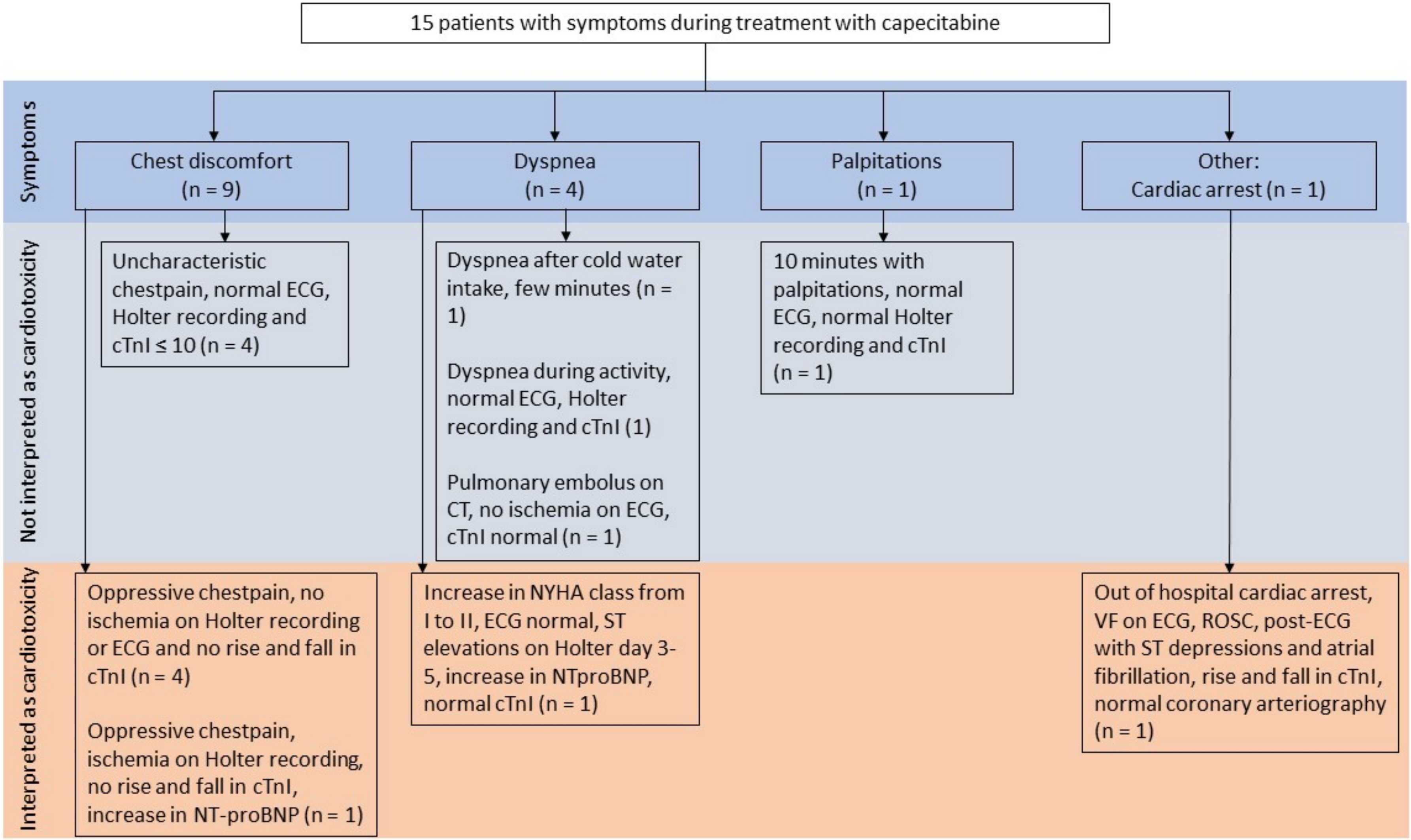

ECG, electrocardiogram; cTnI, cardiac troponin I; NT-proBNP, N-terminal pro-brain-natriuretic peptide; NYHA, New York Heart Association scale; CT, computertomografi scan; ROSC, return of spontaneus circulation.

Table S8: Cardiac troponin I levels in patients with cardiac troponin I above the cut-off level of 40 ng/L during the study period or increases in cardiac Troponin I levels larger than the assay variation but below cut-off level

Case (case no. continued from Table S5)	Before 1st cycle (ng/L)	During 1st cycle (ng/L)	Before 3rd or 4rd cycle (ng/L)	During 3rd or 4rd cycle (ng/L)	Cardiac symptoms	Cardiac comorbidity and risk factors	Prior treatment
**Patients with cardiac troponin I above cut-off level (any timepoint during study period)**							
1 (	10	4620	-	-	Cardiac arrest	Hypertension	Paclitaxel<br>Letrozole<br>Exemestane
6	33	37	42	33	angina during 1st cycle	Aortic valve stenosis (moderate)<br>Hypertension<br>Hypercholesterolaemia	None
13	Missing	4080	4800	4850	No	Active smoker	None
14	57	42	-	-	No	Hypertension<br>Former smoker	None
15	47	25	10	10	No	No	Epirubicin<br>Docetaxel<br>Cyclophosphamide<br>Radiotherapy of left breast
16	10	70	10	10	No	Hypertension<br>Hypercholesterolaemia<br>Former smoker	None
**Patients with increases in cardiac troponin I larger than the assay variation but below the cut-off Level (during either 1st or 3rd/4th cycle)**							
2	10	35	-	-	Acute heart failure and unstable angina	Active smoker	Letrozole<br>Everolimus<br>Exemestane
17	22	27	-	-	No	No	Docetaxel<br>Epirubicin<br>Radiotherapy of left breast<br>Aromatase inhibitor<br>Fulvestrant
18	10	14	-	-	No	Hypertension<br>Former smoker	None
19	16	14	19	26	No	Hypertension<br>Hypercholesterolaemia<br>Former smoker	None
20	11	10	10	17	No	Hypertension<br>Hypercholesterolaemia	None
21	15	10	10	17	No	Hypertension<br>Hypercholesterolaemia<br>Former smoker	None

There were also patients with decreases in cardiac troponin I larger than the assay variation during treatment with capecitabine compared to before (1st cycle: 4 patients; 3rd or 4th cycle: 3 patients).

Figure S4: Median plasma cardiac troponin I levels before and during treatment with capecitabine

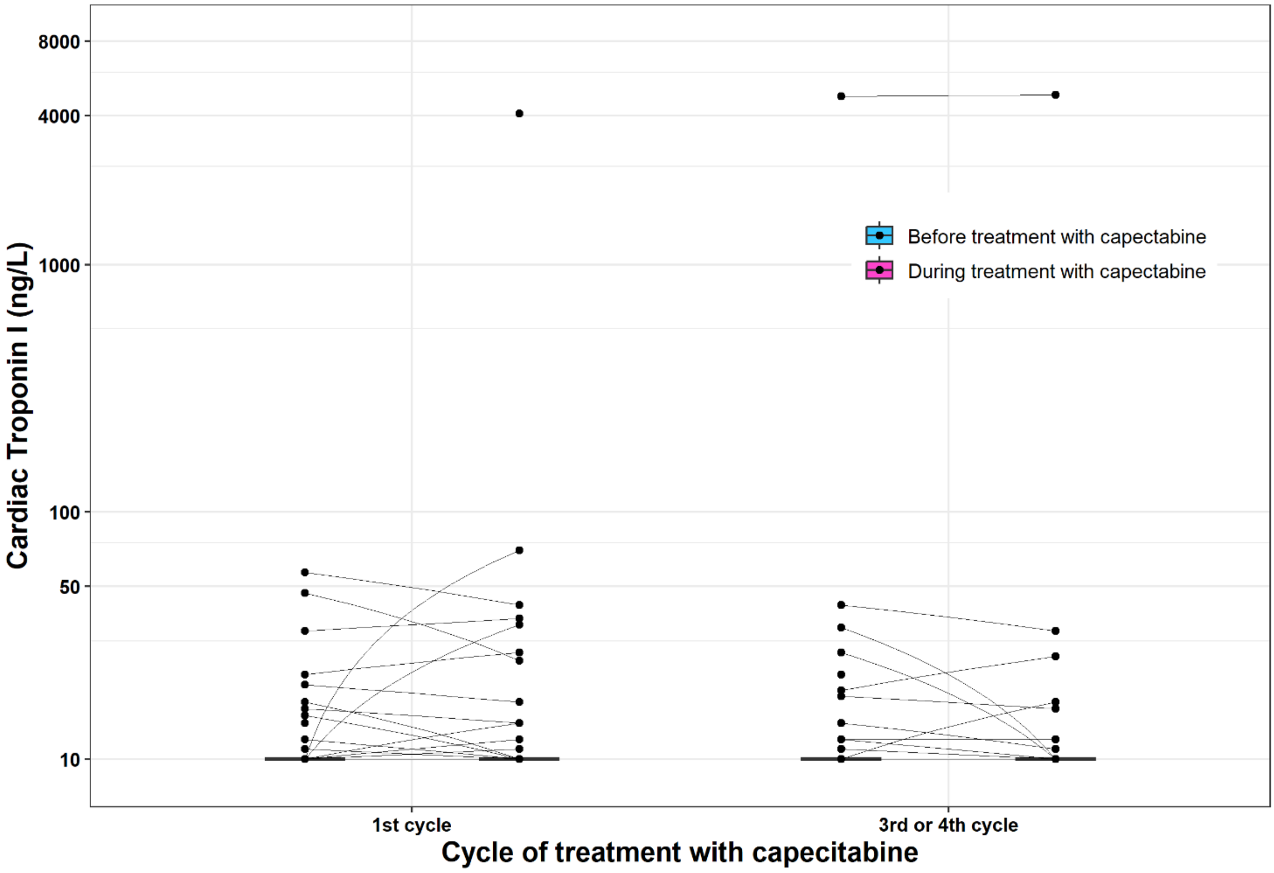

The figure shows the median plasma cardiac troponin I levels (cTnI) before and during treatment with capecitabine in 1st cycle and 3rd or 4th cycle, respectively. The upper and lower boundaries of the boxes (25 and 75 percentiles) cannot be seen since they are equal to the median, because most patients had cTnI values of 10 ng/L (lower detection limit for the assay). The dots represent individual data points, and lines connect datapoints from the patients. There was no difference in plasma cTnI levels before and during treatment in 1st cycle ($p = 0.92$) or 3rd/4th cycle ($p = 0.48$).

Table S9: Number of patients with elevated copeptin before and during treatment

Before 1st cycle	During 1st cycle	Before 3rd or 4rd cycle	During 3rd or 4rd cycle
16 (20.5%)	18 (23.4%)	11 (18.6%)	13 (23.2%)

Table S10: Number of patients with episodes of non-sustained ventricular tachycardia before and during treatment with capecitabine

	Non-sustained ventricular tachycardia before capecitabine	Non-sustained ventricular tachycardia during capecitabine	P-value for difference
1st cycle	6 (7.4%)	16 (19.8%)	**0.021**
3rd or 4th cycle	4 (6.9%)	11 (19.0)	0.065

Table S11: Non-sustained ventricular tachycardia according to the different days in cycle 1: P-values from Wilcoxon signed rank test without and with Bonferroni correction

	p-value (not corrected for multiple comparisons)	p-value (Bonferroni corrected)
Day 1 *v* 0	0.742	1.000
Day 2 *v* 0	0.952	1.000
Day 3 *v* 0	0.497	1.000
Day 4 *v* 0	0.071	0.355
Day 5 *v* 0	0.066	0.330

P values in bold are significant

Discussion of the differences in study design of this study compared to our previous study of 5-FU

In the main manuscript is discussed that the incidence of myocardial ischemia from capecitabine of 7% is lower than the incidence we have previously found for 5-FU of 18.7% [10]. One reason for this difference could be that capecitabine is converted to 5-FU mainly in the tumor tissue and therefore the plasma levels of 5-FU is lower during capecitabine treatment compared to intravenous 5-FU treatment. However, there are also methodological differences between our two studies. Due to the administration of capecitabine for 14 days, it was not possible to monitor the patients with Holter recording throughout the entire treatment period. Thus, myocardial ischemia with debut beyond the days of Holter recording was not detected in the present study. Oppositely, the longer Holter recording time in the present study (7 days) compared to the 5-FU study (3-4 days) increases the likelihood of capturing myocardial ischemia. It is well-known that a day-to-day variation in the number and duration of ischemic episodes on Holter recording is present in patients with chronic ischemic heart disease [11, 12] and therefore a longer recording time may by chance reveal more ischemic episodes due to underlying, unrecognized ischemic heart disease. Furthermore, both studies showed a considerable day-to-day variation in myocardial ischemia during treatment, suggesting that fluctuations in the amount of myocardial ischemia occur in patients with fluoropyrimidine-induced myocardial ischemia.

Alternative treatment options and S-1

Alternative treatment options for patients experiencing capecitabine cardiotoxicity vary based on the type of cancer being treated. For patients with metastatic breast cancer, the next line of treatment is typically initiated [13]. In patients with colorectal or gastric cancer, alternative options may include the oral fluoropyrimidine, S-1. S-1 combines a dihydropyrimidine dehydrogenase (DPD) inhibitor with the 5-FU prodrug, tegafur [14]. The inhibition of DPD reduces the degradation of 5-FU to fluorobetaalanine [14]. Since fluorobetaalanine is sought to play a key role in fluoropyrimidine cardiotoxicity [15], S-1, may be less cardiotoxic compared to 5-FU and capecitabine. S-1 is mainly used in Asian populations [16], but in western populations, S-1 has shown comparable efficacy to capecitabine in metastatic colorectal cancer [17] and to 5-FU in advanced gastric cancer [18]. The data on cardiotoxicity from S-1 are relatively sparse, however, in 200 patients with cardiotoxicity from 5-FU or capecitabine, a switch to S-1 only resulted in recurrent cardiotoxicity in 4% [19]. Also, a small study involving 59 patients with colorectal cancer

compared the cardiotoxicity of capecitabine and S-1 [20]. The results revealed a greater increase in mean daily ischemic burden on Holter recording and higher levels of fluorobetaalanine in patients treated with capecitabine compared to those receiving S-1 [20].

References to the supplementary material

[1] Nordestgaard BG, Langsted A, Mora S, Kolovou G, Baum H, Bruckert E, et al. Fasting Is Not Routinely Required for Determination of a Lipid Profile: Clinical and Laboratory Implications Including Flagging at Desirable Concentration Cutpoints-A Joint Consensus Statement from the European Atherosclerosis Society and European Federation of Clinical Chemistry and Laboratory Medicine. Clin Chem. 2016;62(7):930-46. https://doi.org/10.1373/clinchem.2016.258897

[2] Rydén L, Grant PJ, Anker SD, Berne C, Cosentino F, Danchin N, et al. ESC Guidelines on diabetes, pre-diabetes, and cardiovascular diseases developed in collaboration with the EASD: the Task Force on diabetes, pre-diabetes, and cardiovascular diseases of the European Society of Cardiology (ESC) and developed in collaboration with the European Association for the Study of Diabetes (EASD). Eur Heart J. 2013;34(39):3035-87. https://doi.org/10.1093/eurheartj/eht108

[3] Byrne RA, Rossello X, Coughlan JJ, Barbato E, Berry C, Chieffo A, et al. 2023 ESC Guidelines for the management of acute coronary syndromes. Eur Heart J. 2023;44(38):3720-826. https://doi.org/10.1093/eurheartj/ehad191

[4] Bjerregaard P, El-Shafei A, Kotar SL, Labovitz AJ. ST segment analysis by Holter Monitoring: methodological considerations. Ann Noninvasive Electrocardiol. 2003;8(3):200-7.

[5] Apple FS, Sandoval Y, Jaffe AS, Ordonez-Llanos J. Cardiac troponin assays: Guide to understanding analytical characteristics and their impact on clinical care. Clin Chem. 2017;63(1):73-81. https://doi.org/10.1373/clinchem.2016.255109

[6] Product Specifications: Thermo Scientific BRAMS Copeptin proAVP: Thermo Fischer Scientific; [cited 2025 May 27]. Available from: https://www.copeptin.com/images/downloads/pdf/data-sheet-copeptin-proavp-kryptor.pdf.

[7] Mueller C, Möckel M, Giannitsis E, Huber K, Mair J, Plebani M, et al. Use of copeptin for rapid rule-out of acute myocardial infarction. Eur Heart J Acute Cardiovasc Care. 2018;7(6):570-6. https://doi.org/10.1177/2048872617710791

[8] Katritsis DG, Zareba W, Camm AJ. Nonsustained ventricular tachycardia. J Am Coll Cardiol. 2012;60(20):1993-2004. https://doi.org/10.1016/j.jacc.2011.12.063

[9] QT interval and drug therapy. BMJ. 2016;353:i2732. https://doi.org/10.1136/bmj.i2732

[10] Dyhl-Polk A, Schou M, Vistisen KK, Sillesen AS, Serup-Hansen E, Faber J, et al. Myocardial Ischemia Induced by 5-Fluorouracil: A Prospective Electrocardiographic and Cardiac Biomarker Study. Oncologist. 2021;26(3):e403-e13. https://doi.org/10.1002/onco.13536

[11] Forslund L, Hjemdahl P, Held C, Eriksson SV, Bjorkander I, Rehnqvist N. Prognostic implications of ambulatory myocardial ischemia and arrhythmias and relations to ischemia on exercise in chronic

stable angina pectoris (the Angina Prognosis Study in Stockholm [APSIS]). Am J Cardiol. 1999;84(10):1151-7. https://doi.org/10.1016/s0002-9149(99)00526-3

[12] Sajadieh A, Nielsen OW, Rasmussen V, Hein HO, Hansen JF. Prevalence and prognostic significance of daily-life silent myocardial ischaemia in middle-aged and elderly subjects with no apparent heart disease. Eur Heart J. 2005;26(14):1402-9. https://doi.org/10.1093/eurheartj/ehi169

[13] Gennari A, Andre F, Barrios CH, Cortes J, de Azambuja E, DeMichele A, et al. ESMO Clinical Practice Guideline for the diagnosis, staging and treatment of patients with metastatic breast cancer. Ann Oncol. 2021;32(12):1475-95. https://doi.org/10.1016/j.annonc.2021.09.019

[14] Malet-Martino M, Jolimaitre P, Martino R. The prodrugs of 5-fluorouracil. Curr Med Chem Anticancer Agents. 2002;2(2):267-310.

[15] Polk A, Vistisen K, Vaage-Nilsen M, Nielsen DL. A systematic review of the pathophysiology of 5-fluorouracil-induced cardiotoxicity. BMC Pharmacol Toxicol. 2014;15:47. https://doi.org/10.1186/2050-6511-15-47

[16] Garcia-Alfonso P, Munoz Martin AJ, Ortega Moran L, Soto Alsar J, Torres Perez-Solero G, Blanco Codesido M, et al. Oral drugs in the treatment of metastatic colorectal cancer. Ther Adv Med Oncol. 2021;13:17588359211009001. https://doi.org/10.1177/17588359211009001

[17] Kwakman JJM, Simkens LHJ, van Rooijen JM, van de Wouw AJ, Ten Tije AJ, Creemers GJM, et al. Randomized phase III trial of S-1 versus capecitabine in the first-line treatment of metastatic colorectal cancer: SALTO study by the Dutch Colorectal Cancer Group. Ann Oncol. 2017;28(6):1288-93. https://doi.org/10.1093/annonc/mdx122

[18] Ajani JA, Rodriguez W, Bodoky G, Moiseyenko V, Lichinitser M, Gorbunova V, et al. Multicenter phase III comparison of cisplatin/S-1 with cisplatin/infusional fluorouracil in advanced gastric or gastroesophageal adenocarcinoma study: the FLAGS trial. J Clin Oncol. 2010;28(9):1547-53. https://doi.org/10.1200/JCO.2009.25.4706

[19] Osterlund PJ, Kinos S, Halonen P, Soveri L-M, Kwakman JJ, Salminen T, et al. Feasibility of switching to S-1 after other fluoropyrimidine-related cardiotoxicity during chemotherapy for solid tumors. Journal of Clinical Oncology. 2020;38(15_suppl):7037-. https://doi.org/10.1200/JCO.2020.38.15_suppl.7037

[20] Clive S, Bularga A, Henriksen P, Newby DE, Wall LR, Dawson L, et al. 2157P A phase II randomised controlled trial comparing the cardiotoxicity of capecitabine and S-1. Annals of Oncology. 2023;34:S1122. https://doi.org/10.1016/j.annonc.2023.09.939
